# A nomogram model for screening the risk of diabetes in a large-scale Chinese population: an observational study from 345,718 participants

**DOI:** 10.1038/s41598-020-68383-7

**Published:** 2020-07-14

**Authors:** Mingyue Xue, Yinxia Su, Zhiwei Feng, Shuxia Wang, Mingchen Zhang, Kai Wang, Hua Yao

**Affiliations:** 1 College of Public Health, Xinjiang Medical University, Ürümqi, 830011 China; 2 Center of Health Management, The First Affiliated Hospital, Xinjiang Medical University, Ürümqi, 830011 China; 3 College of Basic Medicine, Xinjiang Medical University, Ürümqi, 830011 China; 4 The First Affiliated Hospital of Xinjiang Medical University, Ürümqi, 830011 China; 5 College of Medical Engineering and Technology, Xinjiang Medical University, Ürümqi, 830011 China

**Keywords:** Computational biology and bioinformatics, Endocrinology

## Abstract

Our study is major to establish and validate a simple type||diabetes mellitus (T2DM) screening model for identifying high-risk individuals among Chinese adults. A total of 643,439 subjects who participated in the national health examination had been enrolled in this cross-sectional study. After excluding subjects with missing data or previous medical history, 345,718 adults was included in the final analysis. We used the least absolute shrinkage and selection operator models to optimize feature selection, and used multivariable logistic regression analysis to build a predicting model. The results showed that the major risk factors of T2DM were age, gender, no drinking or drinking/time > 25 g, no exercise, smoking, waist-to-height ratio, heart rate, systolic blood pressure, fatty liver and gallbladder disease. The area under ROC was 0.811 for development group and 0.814 for validation group, and the *p* values of the two calibration curves were 0.053 and 0.438, the improvement of net reclassification and integrated discrimination are significant in our model. Our results give a clue that the screening models we conducted may be useful for identifying Chinses adults at high risk for diabetes. Further studies are needed to evaluate the utility and feasibility of this model in various settings.

## Introduction

Diabetes, as a group of metabolic disorders characterized by hyperglycemia, can lead to many serious, long-term complications^1–3^. The global epidemic of diabetes currently affects more than 440 million people. The Asia-pacific region has the highest number of people with diabetes, and the prevalence of diabetes in this region has risen sharply in recent decades^4–6^. With a population of 1.38 billion and about 110 million people with diabetes, China now has the largest number of diabetes in the world^7^ and the number continues to grow, placing a huge burden on the health care system. In the 2013 study, which included 170,287 participants, the prevalence of diabetes was 10.9 percent, with 60 percent not knowing they had been diagnosed with diabetes. In addition, an additional 35.7% of the population found abnormal glucose homeostasis, highlighting the large number of people at risk for Diabetes^8^. The reasons for the missed diagnosis are on the one hand the lack of self-awareness of disease management, on the other hand, it is caused by the inaccuracy of diabetes results (only by checking the fasting blood glucose)^9,10^. The ideal way is to check the fasting blood glucose and the two-hour blood glucose value after the oral glucose tolerance test (OGTT) at the same time. However, universal access to blood sugar testing seems unlikely in Northwest China, where the medical standards are poor.

The prevalence of diabetes in Xinjiang is at a high level. According to the 2018 national health examination data of Xinjiang, the detection rate in Urumqi is the highest, reaching 13.9%. Research has proved that a healthy lifestyle and a reasonable diet structure can effectively delay or prevent the occurrence of type||diabetes mellitus (T2DM)^11,12^. At present, most of the diabetes risk prediction models are based on the western white population^13–15^, but research shows that the diabetes risk prediction model based on the white population is not suitable for other ethnicities^16^. The diabetes risk prediction models previously established in China are mostly concentrated in southern regions with flourishing economy^17,18^. A nomogram is widely used for conditions such as gastric cancer, invasive ductal carcinoma, prostate cancer, and osteosarcoma^19–21^, its application in the field of T2DM is relatively small.

The objective of this study is to use non-invasive and directly observable clinical data to construct an intuitive nomogram model to identify patients with increased risk for T2DM. Our approach will be particularly useful in locations with high epidemiological risk and poor socioeconomic status, where patients cannot afford medical laboratory costs.

## Materials and methods

### Study population

Xinjiang physical examination (NJPE) is a large community-based provided by the government which is free for citizens. The data in this study were obtained from the physical examination data of Urumqi, the capital of Xinjiang, in 2018. A total of 643,439 subjects participated in the examination. Subjects who signed a written informed consent were eligible to participate in the study. Potential participants were excluded if they: (1) pregnant; (2) People with type|diabetes mellitus (T1DM); (3) age < 20 years old. With a strict data filtration, 345,718 subjects from the eligible participants were utilized to develop a nomogram model. The data were randomly divided into development group (n = 242,003) and validation group (n = 103,715), with the ratio of 7:3. We have used the data from the development set to build the nomogram, and used the data from the validation set to verify the model. This study was performed in accordance with the principles outlined in the Declaration of Helsinki and approved by the CDC ethical committee and the institutional review board.

### Diagnosis of diabetes

The definition of diabetes in this study was: 2 h after meal blood glucose ≥ 11.1 mmol/l, fasting blood glucose ≥ 7.0 mmol/l, or the main complaint of diabetes and taking hypoglycemic drugs.

### Variable characteristics

NJPE variables include three parts: questionnaire, physical examination and laboratory testing. The questionnaire includes information on medical history and lifestyle, such as smoking, drinking, diet and exercise habits. Physical measurement indexes include height, body weight, heart rate, blood pressure, waist circumference and B-ultrasonic. Laboratory test indicators include blood glucose and blood biochemistry examination. In this study, we wanted to establish a simple model that can predict the risk of T2DM, so this study didn’t include the blood sampling.

### Variable definitions

The potential risk factors in this study to assess T2DM included: age, gender, ethnicity, career, waist-to-height ratio (WHtR), heart rate, diastolic blood pressure (DBP), systolic blood pressure (SBP), exercise situation, smoking amount, drinking amount and some comorbidities.

Basic information of participants: gender included "male" and "female"; career included "trader or service people", "agriculture workers", "factory workers", "soldier" and "others"; ethnic groups were divided into six categories: "Han", "Uygur", "Kazak", "Hui", "Mongolian" and "other nationalities"; WHtR was obtained by dividing waist (cm) by the height (cm); the baseline comorbidities considered in this study were mental diseases, eye diseases, fatty liver and gallbladder disease: defined as yes and no. The presence of eye diseases was defined as diagnosis of one or more of the following: retinal hemorrhage, papilledema and cataract. The diagnosis of gallbladder disease includes cholecystitis, cholecystectomy and cholecystolithiasis.

In these diseases’ history, the diagnosis of mental diseases and eye diseases was mainly based on the self-report of the patients, and the diagnosis of fatty liver and galltesticles disease was made by B-scan. The diagnosis of fatty liver was determined by the professionals of various physical examination institutions according to the standard of China Association of liver diseases^22^. Patients are diagnosed with fatty liver if they meet the following two diagnostic criteria: subjects without specific diseases which can lead to fatty liver such as viral hepatitis, drug-induced liver disease, total parenteral nutrition, hepatolenticular degeneration, and autoimmune liver disease; and liver imaging of subjects was consistent with the diagnostic criteria for diffuse fatty liver.

The questionnaire showed us the living habits of the participants. Living habits included smoking, drinking and exercise. Exercise included single exercise time (minutes), and exercise situation (“yes” or “no”); Smoking amount (cigarettes) were divided into three categories: “no smoking”, “somking/day 0–20 cigarettes” and “somking/day > 20 cigarettes”; drinking amount (g) were divided into three categories: “no drinking”, “drinking/time 0–25 g” and drinking/time > 25 g”.

### Statistical analysis

NJPE data are large, and with jumbled variables, existing some missing and abnormal values. So data pre-processing is a very important step, the quality of preprocessing will directly affect the performance of later prediction model^23^. Firstly, we deleted nearly 200 variables that were not meaningful to this study. Secondly, we defined the outliers as null values and graphically show the absence of data. Thirdly, we filled in nulls, classification variables were filled with the most frequent value, and continuous variables were filled with mean value.

When it comes to the prevalence rate of diabetes, we calculated age-standardised and sex-standardised rates of T2DM prevalence, using data from all 31 provinces in the 2018 Chinese census^24^. We assigned individuals different weights so that the age and sex distributions matched the census data.

While comparing the baseline characters between the development group and validation group, the chi-square test is used for categorical variables, and t-test or Wilcoxon rank sum test for quantitative variables. The least absolute shrinkage and selection operator (LASSO) regression was used to screen the risk factors. LASSO combines the advantages of selection process (easy to explain) and expression (robust), which is particularly useful in large data sets requiring efficient and fast algorithms^25,26^. First, features with nonzero coefficients in the LASSO regression model were selected. Then, multivariable logistic regression analysis was used to build a predicting model by incorporating the feature selected in the LASSO regression model. The features were considered as odds ratio (OR) having 95% confidence interval (CI) and as *p*-value. The statistical significance levels were all two sided. Variables with the *p*-value of 0.05 were included in the model^19,27^. Multicollinearity test were performed since we included quite a few variables as covariates.

We used two methods to assess the proposed nomogram. First, receiver operation characteristic curve (ROC) was used to evaluate the discrimination. The value of area under curve (AUC) exists between 0.5 and 1. A value of the AUC closer to 1 indicates a good performance of the predictive model^28^. Second, Hosmer–Lemeshow good of fit test was used to evaluate the calibration. If the smaller the chi square value of the statistics is, the larger the corresponding *p *value is, the better the calibration of the prediction model will be. If the test results show statistical significance (*p* < 0.05), it shows that there is a certain difference between the predicted value of the model and the actual observed value, and the model calibration is poor^29^. Also, by calculating the net reclassification improvement (NRI) and integrated discrimination improvement (IDI), we could calculate the improvement of accuracy compared with the previous model^30^. In addition, we established a prediction model for T2DM by gender. In the appendix lists the related risk factors of men and women of multivariate logistic regression analysis as a result, and their nomogram model and model validation (Supplementary Table S1; Supplementary Figs. S1, S2).

The open source Python software Version 3.7.2 (https://www.python.org) was used for data pre-processing, Pandas library and NumPy library were used for interpolation and processing of outliers, and Matplotlib library was used for data description and outliers judgment; the open source R software Version 3.6.1 (https://www.r-project.org) was used for data modeling, CARET-package was used to divide the data into development and validation group randomly, LASSO analysis was performed with glmnet-packages, RMS-package was used to establish a nomogram model, the nomogramEx-package was used to figure the scores of every characters in nomogram, the ROC was plotted using ROCR-package and PROC-package, and the calibration curves had been drawn by RMS-package.

## Results

### The missing data of covariates

**Figure 1:**
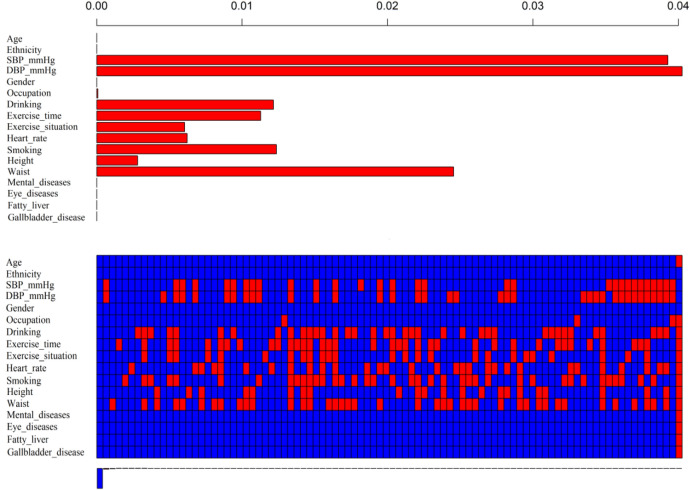
Missing data of covariates (Figure was created by R software, VIM-package Version 4.7.0, https://CRAN.R-project.org/package=VIM).

Our research has included 17 covariables, Fig. 1. shows for us about all covariate loss, the above bar chart shows the proportion of different covariate data missing, and the top of X-axis corresponds to the missing proportion, which can be seen that the variable DBP has the largest missing proportion (4%). In addition, age, ethnicity, gender, mental diseases, eye diseases, fatty liver, and gallbladder disease have no missing values. The graph below shows the missing data in red and no missing data in blue. In this part of the figure, the composition of the missing values of each sample can be obtained, as the corresponding X-axis at the bottom represents the proportion of different missing composition structures of the samples. For example, among all the samples, the proportion of the samples without missing values is the largest, and samples only missing SBP and DBP accounts for the second.

### Characteristics of physical examination population

A total of 345,718 cases were included in the study, including 242,003 in the development group, 103,715 in the validation group. In our data, the T2DM prevalence was 13.96%, while in development set and validation set were 13.95% and 13.96%, respectively. The age-standardised and sex-standardised rates of T2DM prevalence was 9.80%, while in development set and validation set were 9.81% and 9.79%, respectively. All data of cases including demographic and disease in the two groups were given in Table 1. The comparison of baseline data showed that there was no significant difference between the development group and the validation group.

**Table 1:** Characteristics of the development set and validation set, data are shown as means ± SD, or no.

Characteristic	Development set (n = 242,003)	Validation set (n = 103,715)	p value
Age (years)	53.89 ± 15.93	53.88 ± 15.89	0.059
Heart rate	75.69 ± 9.20	75.65 ± 9.18	0.213
DBP (mmHg)	75.83 ± 10.12	75.81 ± 10.11	0.569
SBP (mmHg)	124.60 ± 16.11	124.60 ± 16.02	0.986
Ethnicity, n (%)			0.788
Han	161,541 (66.8)	69,300 (66.8)	
Uygur	39,569 (16.4)	17,051 (16.4)	
Kazak	7,270 (3.0)	3,036 (2.9)	
Hui	28,048 (11.6)	11,953 (11.5)	
Mongolian	469 (0.2)	208 (0.2)	
Other nationalities	5,106 (2.1)	2,167 (2.1)	
WHtR			0.113
< 0.4	4,581 (1.9)	1,875 (1.8)	
0.4–0.5	81,024 (33.5)	35,036 (33.8)	
0.5–0.6	123,270 (50.9)	52,757 (50.9)	
> 0.6	33,127 (13.7)	14,046 (13.5)	
Gender, n (%)			0.712
Male	112,137 (46.3)	48,130 (46.4)	
Female	129,866 (53.7)	55,585 (53.6)	
Career			0.732
Trader or service people	170,773 (70.6)	73,380 (70.8)	
Agriculture workers	53,716 (22.2)	22,917 (22.1)	
Factory workers	6,424 (2.7)	2,688 (2.6)	
Soldier	1,429 (0.6)	598 (0.6)	
Others	9,661 (4.0)	4,132 (4.0)	
**Exercise**			
Single exercise time (min)	18.04 ± 29.69	18.09 ± 29.77	0.656
Exercise situation, n (%)			0.694
Yes	82,156 (33.9)	35,282 (34.0)	
No	159,847 (66.1)	68,433 (66.0)	
Drink amount (g), n (%)			0.362
No drinking	213,210 (88.1)	91,461 (88.2)	
0–25 g per time	25,778 (10.7)	10,920 (10.5)	
> 25 g per time	3,015 (1.2)	1,334 (1.3)	
Smoking amount (cigarettes), n (%)			0.050
No smoking	213,489 (88.2)	91,952 (88.5)	
0–20 cigarettes per day	26,402 (10.9)	10,892 (10.7)	
> 20 cigarettes per day	2,112 (0.9)	871 (0.8)	
Mental diseases, n (%)			0.915
Yes	6,640 (2.7)	2,839 (2.7)	
No	235,363 (97.3)	100,876 (97.3)	
Eye diseases, n (%)			0.510
Yes	9,044 (3.7)	3,926 (3.8)	
No	232,959 (96.3)	99,839 (96.2)	
Fatty liver, n (%)			0.754
Yes	46,427 (19.2)	19,849 (19.1)	
No	195,576 (80.8)	83,866 (80.9)	
Gallbladder disease, n (%)			0.856
Yes	23,757 (9.8)	10,203 (9.8)	
No	218,246 (90.2)	93,512 (90.2)	

*WHtR* waist-to-height ratio, *DBP* diastolic blood pressure, *SBP* systolic blood pressure.

### Characteristics selection

**Figure 2:**
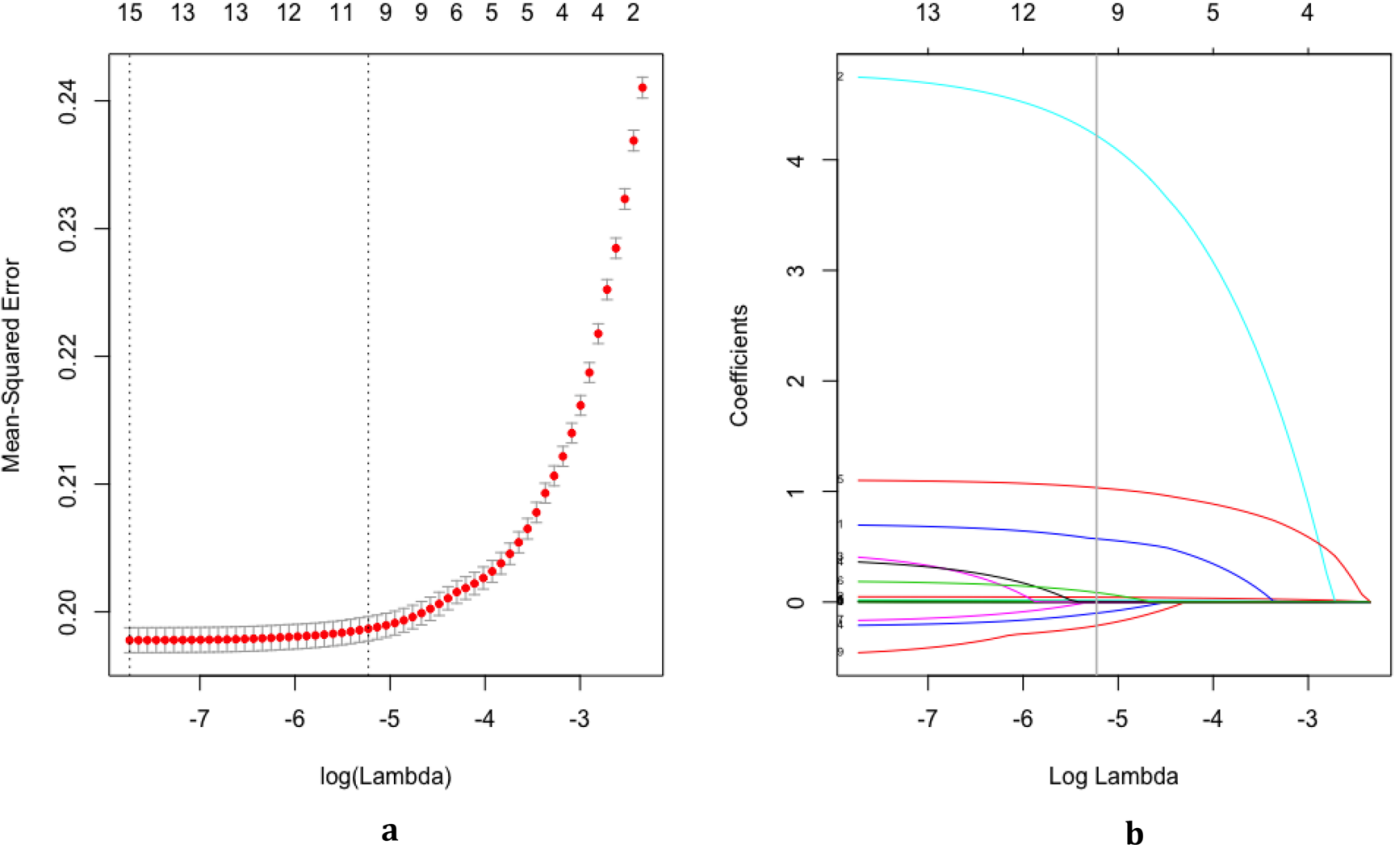
Texture feature selection using the least absolute shrinkage and selection operator (LASSO) binary logistic regression model (Figure was created by R software, glmnet-package Version 2.0-18, https://CRAN.R-project.org/package=glmnet). (**a**) Optimization parameters (lambda) of LASSO model were selected by 10 times cross-validation. The Mean-Squared Error was plotted versus log (Lambda). Dotted vertical lines were drawn at the optimal values by using the minimum criteria and the one standard error of the minimum criteria (the 1-SE criteria). Minimum criteria refer to the one among all λ values to get the mean value of the minimum target parameter. 1-SE criteria refer to the λ value of the simplest model in a variance range of minimum criteria. (**b**) LASSO coefficient profiles of the 16 features. A coefficient profile plot was produced against the log (Lambda) sequence. Vertical line was drawn at the value selected using 10 times cross-validation, where optimal lambda resulted in 10 features with nonzero coefficients.

Through LASSO regression, we got 10 non-zero coefficient characteristics, which showed that we reduced 16 indexes to 10 indexes. As it was shown in Figs. 2a, b. These features included age, gender, ethnicity, drinking amount (g), exercise situation, smoking amount (cigarettes), heart rate, WHtR, fatty liver and gallbladder disease (Table 2).

**Table 2:** Multivariate logistic regression analysis for risk factors associated T2DM in the development set (N = 242,003).

Intercept and variable	β	Odds ratio	95% CI	Z value	p value
Age	0.048	1.049	(1.048–1.050)	80.332	< 0.001
Heart rate	0.013	1.013	(1.011–1.014)	15.949	< 0.001
SBP	0.017	1.017	(1.016–1.018)	36.546	< 0.001
**Gender**					
Men	Ref	1	Ref	–	–
Female	–0.294	0.745	(0.721–0.770)	–17.394	< 0.001
**Exercise situation**					
No	Ref	1	Ref	–	–
Yes	–0.350	0.705	(0.682–0.728)	–21.149	< 0.001
**Drinking amount**					
No drinking	Ref	1	Ref	–	–
0–25 g per time	–0.258	0.772	(0.734–0.814)	-9.838	< 0.001
> 25 g per time	0.487	1.627	(1.447–1.827)	8.168	< 0.001
Smoking amount					
No smoking	Ref	1	Ref	–	–
0–20 cigarettes per day	0.388	1.474	(1.401–1.551)	14.951	< 0.001
> 20 cigarettes per day	1.205	3.336	(2.956–3.763)	19.573	< 0.001
**WHtR**					
< 0.4	Ref	1	Ref	–	–
0.4–0.5	0.486	1.626	(1.315–2.037)	4.364	< 0.001
0.5–0.6	1.065	2.901	(2.351–3.627)	9.643	< 0.001
≥ 0.6	1.492	4.445	(3.596–5.566)	13.402	< 0.001
**Fatty liver**					
No	Ref	1	Ref	–	–
Yes	1.127	3.087	(2.987–3.190)	67.350	< 0.001
**Gallbladder disease**					
No	Ref	1	Ref	–	–
Yes	0.197	1.218	(1.167–1.271)	9.023	< 0.001

β is the regression coefficient.

*CI* Confidence interval, *WHtR* waist-to-height ratio, *SBP* systolic blood pressure.

### Independent prognostic factors in the development group

The 10 variables obtained by LASSO regression were included in logistic multiple regression model, and the regression results were shown in Table 2. Among them, the OR greater than 1 is an independent risk factor for the disease, while less than 1 is the protection factor. In addition, there was no evidence of multicol-linearity among the covariates included in the model. Maximum VIF (variance inflation factor) was 1.33, and lowest eigen value was 1.06.

### Nomogram of diabetes

**Figure 3:**
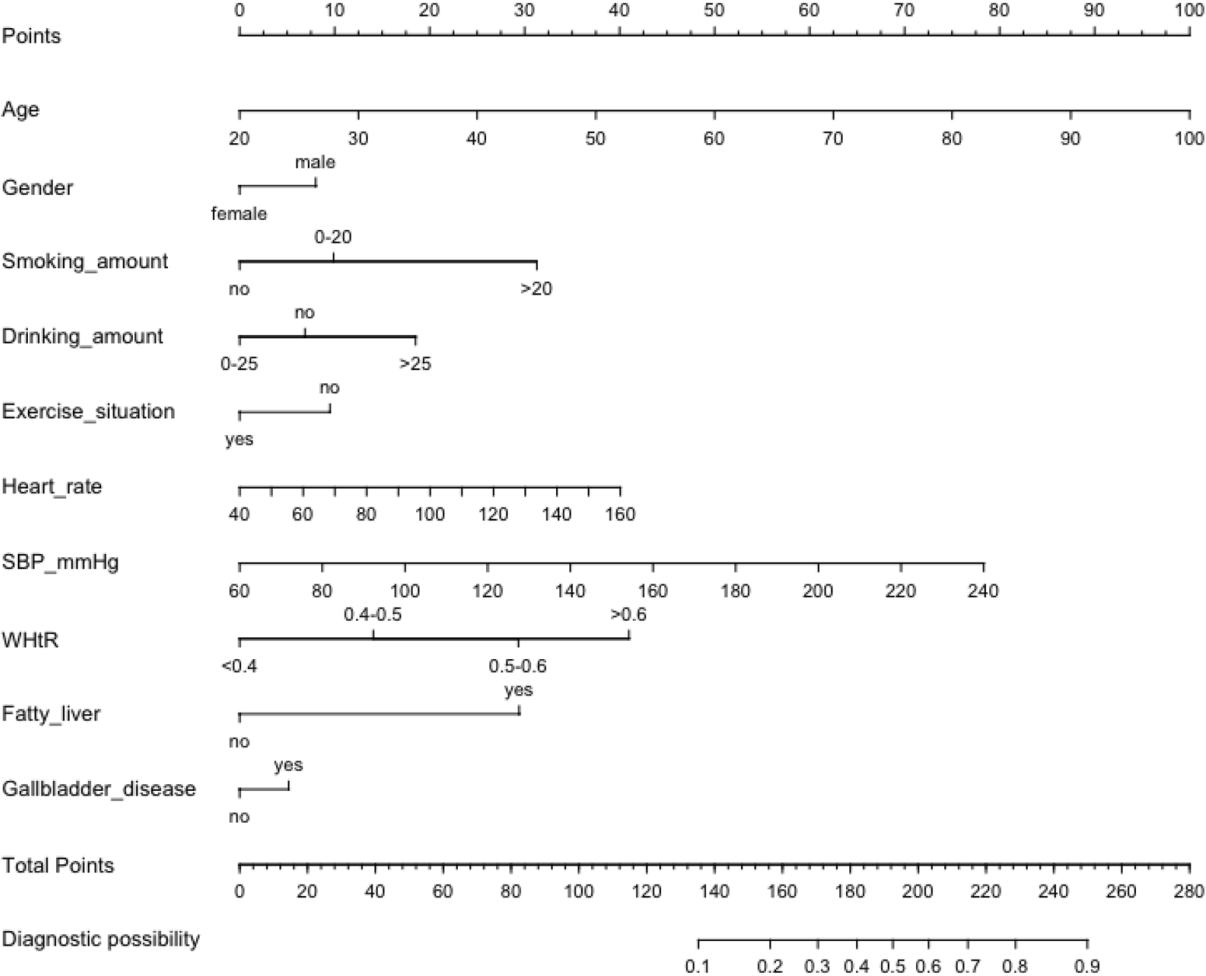
Nomogram to predict the risk of T2DM. *WHtR* Body Mass Index, *SBP* systolic blood pressure (Figure was created by R software, rms-package Version 5.1-3.1, https://CRAN.R-project.org/package=rms). To use the nomogram, an individual participants value is located on each variable axis, and a line is drawn upward to determine the number of points received for each variable value. The sum of these numbers is located on the Total Points axis to determine the risk of T2DM.

Based on logistic multiple regression and LASSO, we got Urumqi Diabetes Nomogram Model consisting of 10 factors (Fig. 3). Each sub-type in these variables is assigned a score. The cumulative sum of each “point” is the “total points”. The corresponding “diagnostic possibility” of “total point” is the predicted probability of T2DM suggested by our designed nomogram.

Take an example of nomogram usage: a sample was randomly selected from the developing set, a 60 year old man, drinking/time > 25 g, no exercise, smoking/day 15 cigarettes, WHtR is 0.58, heart rate is 90 times/min, SBP is 100 mmHg, previous gallbladder disease history, no other history, so his total score was 193.56, and the corresponding possibility of T2DM was close to 50%. We can remind him to stop smoking, control drinking and exercise to prevent diabetes.

### Validation of the nomogram

**Figure 4:**
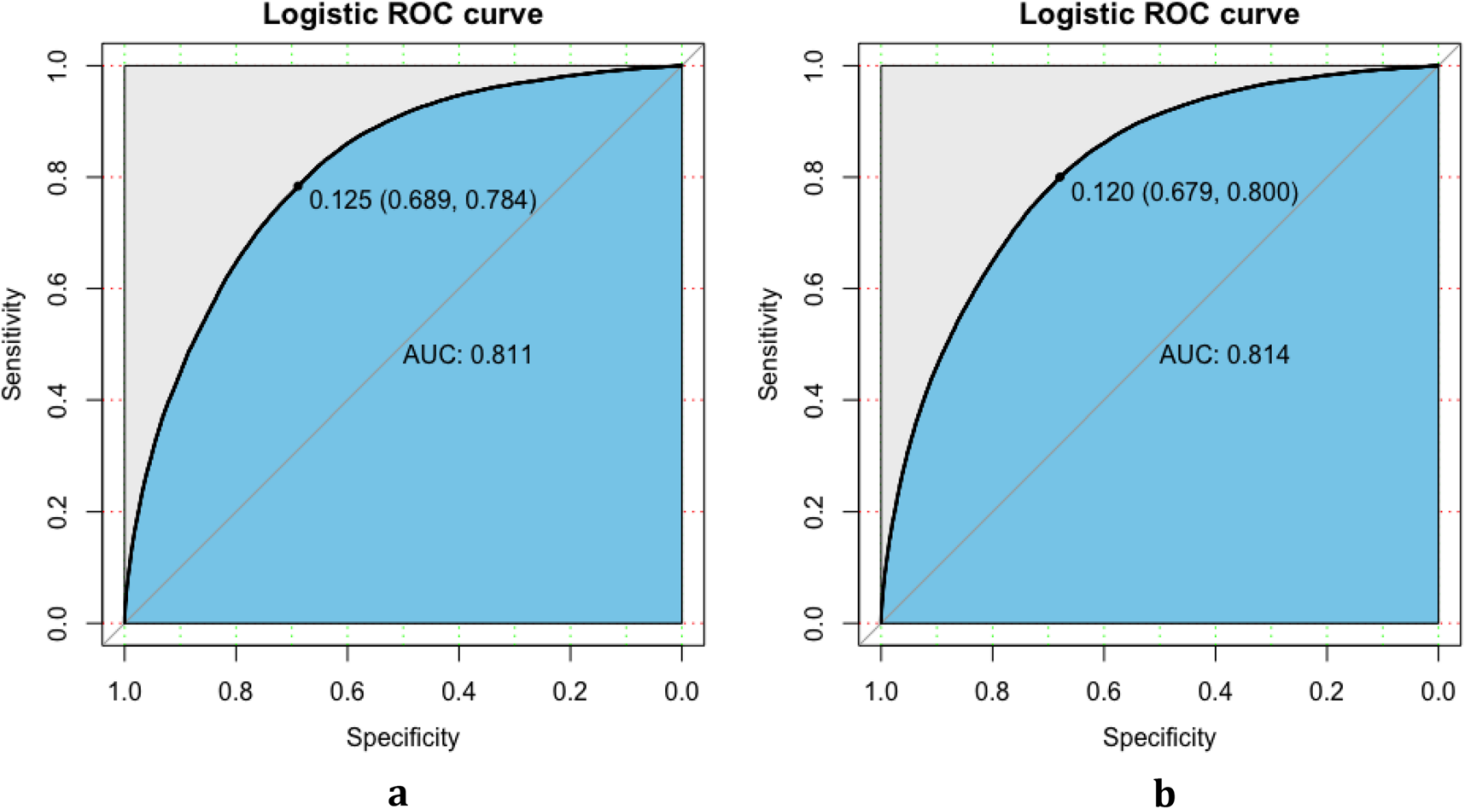
ROC for validating the discrimination power of the nomogram. (**a**) Development group. (**b**) Validation group (AUC = 0.811 vs 0.814) (Figure was created by R software, ROCR-package Version 1.15.3 and PROC-package Version 1.0-7, https://cran.r-project.org/web/packages/ROCR and https://cran.r-project.org/web/packages/pROC).

**Figure 5:**
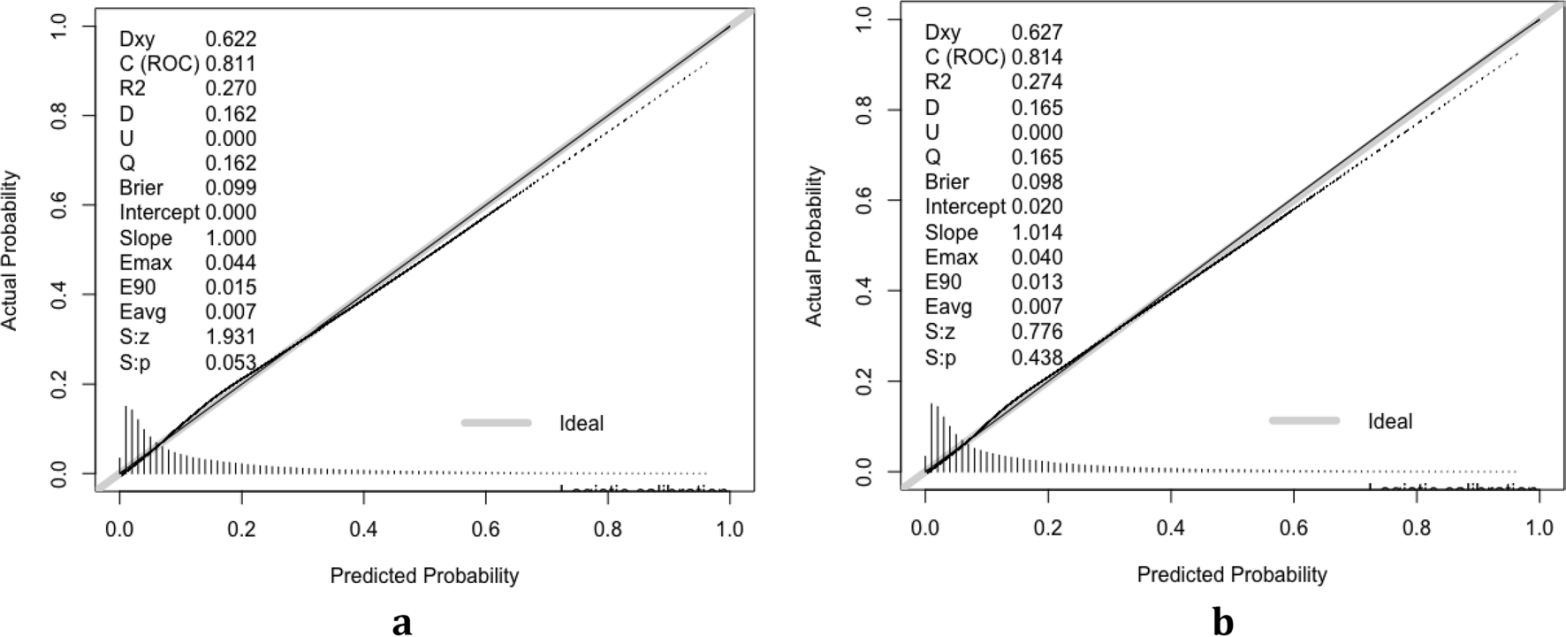
Calibration curves for the validation and development group models (*P* = 0.053 vs 0.438) (Figure was created by R software, rms-package Version 5.1-3.1, https://CRAN.R-project.org/package=rms). The diagonal dotted line represents a perfect prediction by an ideal model. The solid line represents the performance of the nomogram, of which a closer fit to the diagonal dotted line represents a better prediction.

The validation of the model was based on discrimination and calibration. Plot prediction accuracy curve ROC and calculate AUC value of development group and validation group. And the AUC value of development group and validation group was 0.811 and 0.814 respectively (Fig. 4a, b), indicating that nomogram prediction model had good discrimination ability. The calibration of the prediction model was evaluated by Hosmer–Lemeshow good of fit test, and the calibration curve (Fig. 5a, b) was obtained. When *p* > 0.05, the calibration ability of the model is good. The calibration curve of the development group was *p* = 0.053, and the calibration curve of the validation group was *p* = 0.438, all of which were greater than 0.05, indicating that the model had good calibration ability.

In order to asses the clinical usefulness of the model, our model was compared with Thai model^31^, in which gender, age, body mass index (BMI) and SBP were used to classify T2DM. On the Thai model, the AUC of the development set and the validation set of our data were 0.793 and 0.789 respectively. Our model added lifestyle variables and comorbidities to the Thai model, the NRI value were 0.112 (95% CI 0.106–0.117, *p* < 0.001) in the development set and 0.111 (95% CI 0.102–0.121, *p* < 0.001) in the validation set, respectively. Similarly, the IDI values were 0.037 (95% CI 0.36–0.039) in the development set and 0.040 (95% CI 0.037–0.042) in the validation set, respectively. The results show that the classification performance of our new model is better than that of the Thai model.

## Discussion

In this study, based on the NJPE data in Urumqi, Xinjiang, China, a new nomogram was developed to screening of T2DM using simple and easily available variables. Risk factors included age, gender, on drinking or drinking/time > 25 g, no exercise, smoking amount, WHtR, heart rate, SBP, fatty liver and gallbladder disease. In this study, only physical measurement and questionnaire indicators were included, while the blood was not included. So the model can be applied to the pre-diabetes and non-invasive prediction of diabetes without the need for expensive laboratory testing, which is useful, particularly in areas of high epidemiological risk and low socioeconomic status—such as China's northern region of Xinjiang, which has the high prevalence rates of diabetes in the country, and low medical coverage rates^8,32,33^.

In the validation of model, the original data were randomly divided into development set (n = 242,003) and validation set (n = 103,715). The verification results showed good risk prediction ability, AUC of development set and validation set was 0.811 and 0.814, respectively. The calibration plot demonstrated that the constructed nomogram is accurate for predicting the risk of diabetes. In addition, the IDI and NRI showed the newly constructed classification model performed well and it should be used in clinical practice. AUC is an indicator used for the accuracy of classification, when compared with AUC, NRI is prone to the comparison results among models, whose aim is to judge whether the classification effect of one node model has been improved compared with the others. At the same time, the results of NRI are influenced by the threshold of risk categories, the categorization should ideally have clear consequences in clinical practice. When possible, give references to formal clinical guidelines used to define the risk categories for the computation of the NRI^34^ . If alternative cutoffs were used, they need to be clearly explicitly motivated. The NRI should not be interpreted on its own but in the context of complementary statistical measures. If a covariant is not associated with the outcome or does not yield an increase in the AUC, a positive NRI should not be expected^35^.

In terms of diabetes risk factors research, Xu et al.^36^ used the data of national cross-sectional survey in 2010 to study and found that the risk factors of diabetes were age, gender, smoking, overweight, obesity, dyslipidemia, heart rate, elevated triacylglycerol and high systolic blood pressure. Other countries had developed diabetes screening tools. The American Diabetes Association (ADA) provides a simple "T2DM risk test" that used age, gender, family history of diabetes, hypertension, physical activity and weight status to assess diabetes risk in the general population^14^. The above conclusions were consistent with the conclusions of this study, but this study had a large sample size and high accuracy. At the same time, another advantage of this study is the use of nomogram risk prediction chart, which can directly show the degree of diabetes risk in different populations. In terms of nomogram diabetes risk prediction, Seung et al. Of South Korea established nomogram diabetes risk prediction model through national health and nutrition survey data^37^, showing that age, dyslipidemia, cardiovascular disease, family history of diabetes, abdominal obesity, hypertension, male gender, smoking situation, low education level and low income were risk factors of diabetes. However, in addition to age, other indicators were two-classification indexes, such as smoking, exercise and hypertension, which were only divided into two categories (have and have not), so they failed to reflect the impact of frequency and quantity on the disease. Based on the conclusion that smoking was a risk factor, the risk factors continued to increase with the increase of daily smoking. Interestingly, studies have shown that alcohol consumption was a protective factor for T2DM, but increased alcohol consumption increased the risk of diabetes. Previous studies have also shown that light to moderate alcohol consumption could reduce the risk of T2DM^38,39^, however, there was a strong dose–response relationship between smoking number, alcohol consumption and diabetes, cardiovascular disease, and cerebrovascular disease^40–42^, suggesting that while quitting smoking completely and controlling alcohol consumption were our goals, but even smoking fewer cigarettes and drink appropriate amount can reduce the risk of the disease, which was equaled to an old Chinese saying, “A little drink will make you happy and a big drink will hurt you”.

In the prevention of T2DM, we got that exercise, moderate alcohol consumption were protective factors, which was consistent with previous research results^11,12^. The protective factors were both adjustable indicators, which suggested that people could control the occurrence of disease through a good life like.

In the appendix, we established models for both men and women, respectively. In female model, smoking and drinking were not selected by LASSO, therefore they were not the variable having effectively impact on T2MD. We considered this might due to fewer women with the habit of smoking and drinking, so the effect of these two indicators for men were more, this was in line with the actual situation^43,44^. Models based on different genders own good degrees of discrimination and prediction ability, in the development set (AUC = 0.820 for females, AUC = 0.797 for males) and validation set (AUC = 0.816 for females, AUC = 0.796 for males). The AUC values of females were larger than those of males in this study, this result was consistent with some previous similar studies^45,46^.

There are other advantages in our model, first, we used a relatively large community-based sample to set up models which ensures the representativeness and stability. Second, the nomogram is composed of a large number of community residents' physical examination indicators, which are commonly used examination items and can provide good promotion for the rest of the population. Noble et al.^47^ also suggested that sociodemographic and clinical data are much better predictors for the risk of diabetes than genetic markers. Thirdly, the previous models were all classification indicators, and ours included many continuous variables to obtain more accurate predictions. Finally, we used WHtR to replace BMI and waist to construct the model. Previous studies have confirmed that WHtR has a better effect^48^.

Dietary habits as a known determinant for T2DM were not included in this study because they are difficult to accurately assess. At the same time, many people suffer from undiagnosed diabetes^49^, given the inadequacy of China's health system in the last century. As a result, many participants were uncertain about their previous generation's medical history, and family history parameters did not improve risk prediction, so we did not collect data on family history.

There are several limitations in this study: firstly, there is no way to analyze the causal relationship from the cross-sectional data of 2018 national health examination, which needs further verification in the future research. Secondly, the data used in this study is the physical examination data of Urumqi, China, which may limit the extrapolation of the results. It is generally believed that there are some differences in the pathophysiology of diabetes between Asians and Caucasians, and there are similar differences between Asian countries. Therefore, each country needs an appropriate diabetes risk assessment model that reflects the regional characteristics, which will help to effectively prevent diabetes.

## Conclusions

Xinjiang occupies 1/6 land areas of China, and the incidence of diabetes has always been in the forefront. As far as we know, this is the first nomogram study designed for northern China. Nomograms are visual and intuitive, which helps the general population and health managers to undersand the risk of diabetes more easily. In addition, our model has 345,718 pieces of data, which is rare in other previous prediction models, we also got the relationship between the dose of smoking and drinking with diabetes. Therefore, the tool will better predict disease risk and prevent diabetes. In conclusion, we designed nomogram models for Xinjiang people in China, and screened ten risk factors to predict diabetes through LASSO regression and multivariate logistic regression analysis. Our results give a clue that the screening models we conducted may be useful for identifying Chinses adults at high risk for diabetes. Further studies are needed to evaluate the utility and feasibility of this model in various settings.

## Supplementary information

Supplementary information.

## Data Availability

The data used to support the findings of this study are available from the corresponding author upon request.
