# Supplementary material for: A nomogram model for screening the risk of diabetes in a large-scale Chinese population: an observational study from 345,718 participants
Source: Sci Rep. 2020 Jul 14;10:11600. doi: 10.1038/s41598-020-68383-7 (PMC7360758; doi:10.1038/s41598-020-68383-7)
Supplement: Supplementary file 1 — Supplementary information. [file 41598_2020_68383_MOESM1_ESM.docx]

**A Nomogram mode for Screening the Risk of Diabetes in a large-scale Chinese population: an observational study from 345,718 participants**

**Mingyue Xue^1^, Yinxia Su^2^, Zhiwei Feng^3^, Shuxia Wang^2^, Mingchen Zhang^4^, Kai Wang^5*^, Hua Yao^2*^**

**Affiliations:**

^1^ College of Public Health, Xinjiang Medical University, Urumqi 830011, China

^2^ Center of Health Management, The First Affiliated Hospital, Xinjiang Medical University, Urumqi 830011, China

^3^ College of Basic Medicine, Xinjiang Medical University, Urumqi 830011, China

^4^ The first affiliated hospital of Xinjiang medical university, Urumqi 830011, China

^5^ College of Medical Engineering and Technology, Xinjiang Medical University, Urumqi 830011, China

^*^**Correspondence to:**

Kai Wang, PhD

Email: [wangkaimath@sina.com](mailto:wangkaimath@sina.com)

Hua Yao, PhD

Email: yaohua01@sina.com

The variables obtained by LASSO regression for male and female were included in logistic multiple regression model.

| **Intercept and Variable** | **Female** | | | **Male** | | |
| --- | --- | --- | --- | --- | --- | --- |
|  | **Odds Ratio** | **95% CI** | **P value** | **Odds Ratio** | **95% CI** | **P value** |
| Age(years) | 1.050 | (1.049-1.052) | <0.001 | 1.049 | (1.047-1.050) | <0.001 |
| Heart rate | 1.012 | (1.010-1.014) | <0.001 | 1.015 | (1.013-1.016) | <0.001 |
| SBP (mmHg) | 1.017 | (1.016-1.018) | <0.001 | 1.016 | (1.015-1.018) | <0.001 |
| Exercise situation, n (%) |  |  |  |  |  |  |
| No | 1 | Ref | — | 1 | Ref | — |
| Yes | 0.741 | (0.713-0.770) | <0.001 | 0.665 | (0.639-0.692) | <0.001 |
| WHtR |  |  |  |  |  |  |
| <0.4 | 1 | Ref | — | 1 | Ref | — |
| 0.4-0.5 | 1.781 | (1.357-2.392) | <0.001 | 1.798 | (1.397-2.357) | <0.001 |
| 0.5-0.6 | 3.327 | (2.542-4.458) | <0.001 | 3.089 | (2.405-4.041) | <0.001 |
| >=0.6 | 5.123 | (3.908-6.872) | <0.001 | 4.802 | (3.728-6.298) | <0.001 |
| Drink amount (g),n(%) | — | — | — |  |  |  |
| No drinking |  |  |  | 1 | Ref | — |
| 0-25g per time |  |  |  | 0.706 | (0.671-0.743) | <0.001 |
| >25g per time |  |  |  | 1.430 | (1.290-1.584) | <0.001 |
| Smoking amount (cigarettes),n(%) | — | — | — |  |  |  |
| No smoking |  |  |  | 1 | Ref | — |
| 0-20 cigarettes per day |  |  |  | 1.564 | (1.496-1.636) | <0.001 |
| >20cigarettes per day |  |  |  | 4.065 | (3.647-4.529) | <0.001 |
| Fatty liver, n (%) |  |  |  |  |  |  |
| Yes | 1 | Ref | — | 1 | Ref | — |
| No | 2.946 | (2.833-3.063) | <0.001 | 3.254 | (3.128-3.386) | <0.001 |
| Gallbladder disease, n (%) |  |  |  |  |  |  |
| Yes | 1 | Ref | — | 1 | Ref | — |
| No | 1.194 | (1.138-1.253) | <0.001 | 1.234 | (1.168-1.304) | <0.001 |

**Table S1.** Multivariate logistic regression analysis for risk factors associated T2DM in the development group for males and females


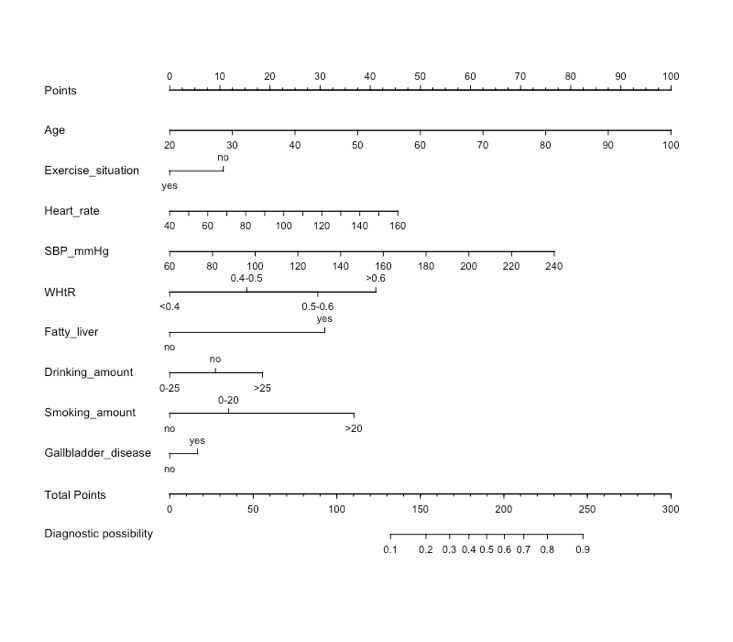

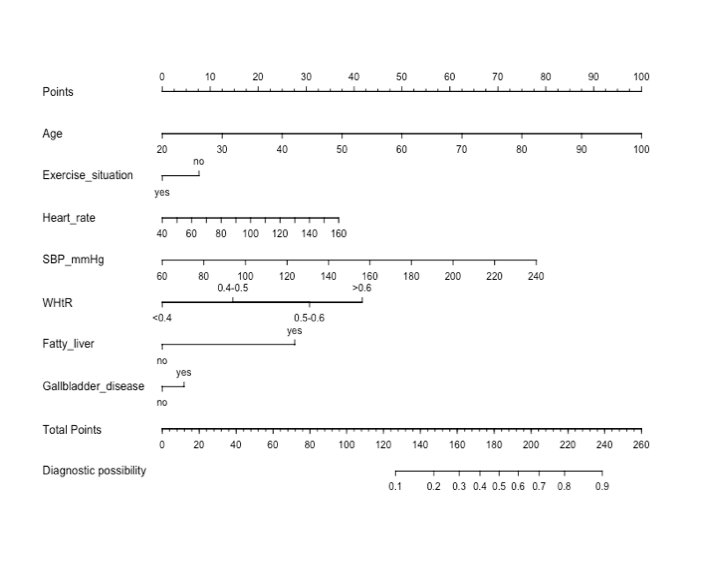


**S1a S2b**

**Fig. S1** Nomogram to predict the risk of T2DM for **a.** males and **b.** females.


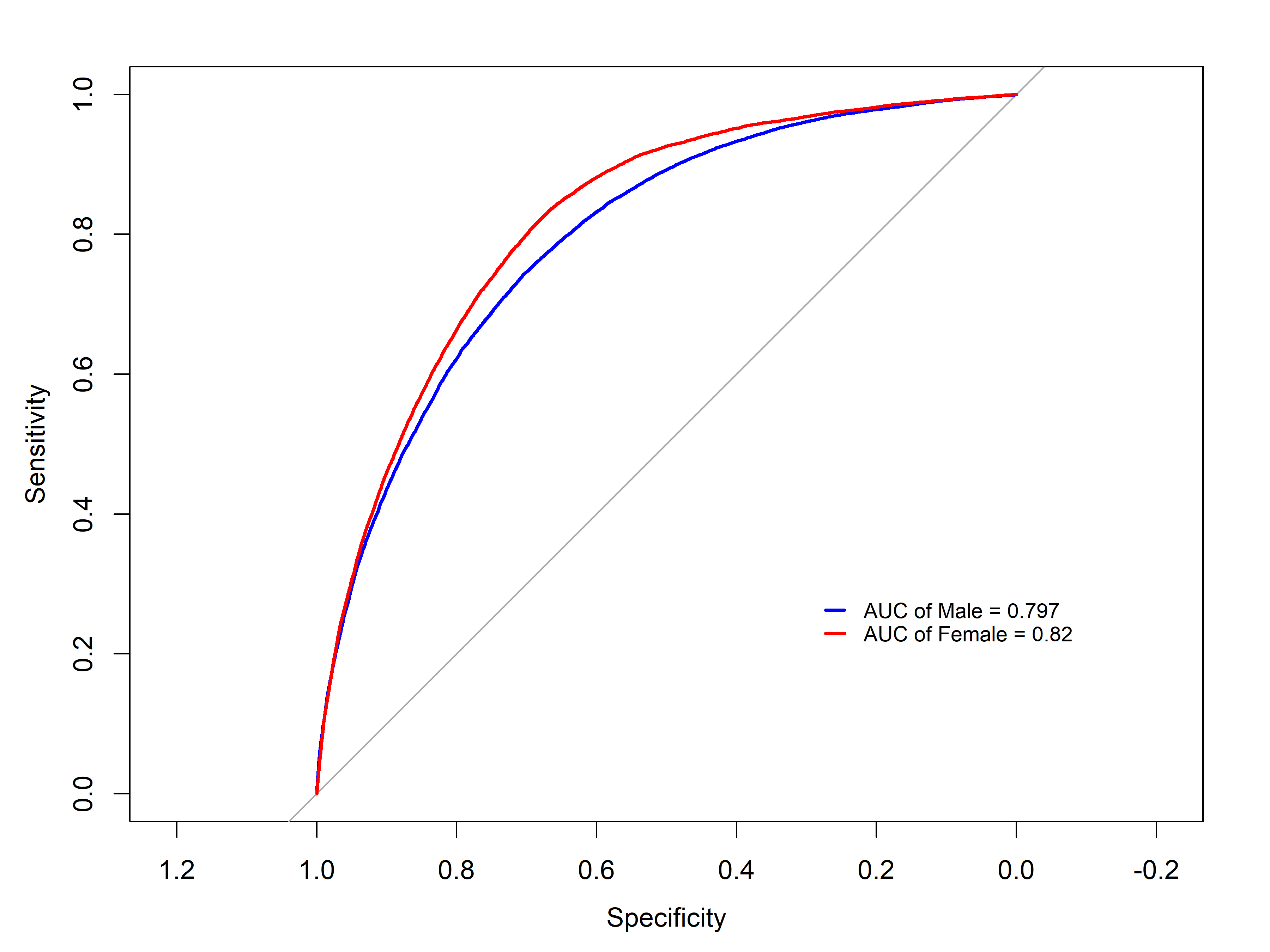

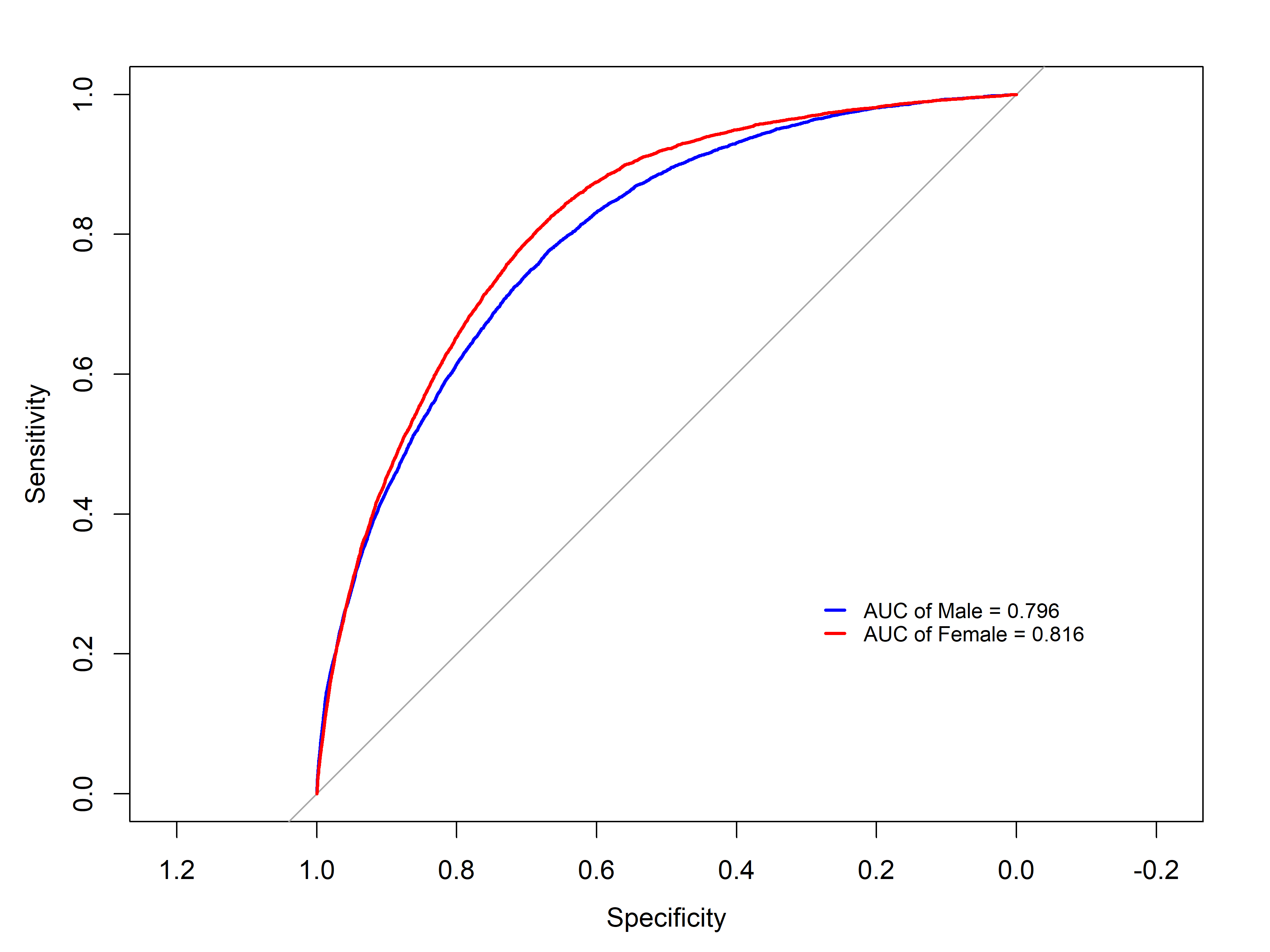


**S2a**   **S2b**

**Fig. S2** The ROC curves of the nomogram for T2DM risk in the **a.** Development group and **b.** Validation group for males and females
